# An advanced bipolar device helps reduce the rate of postoperative pancreatic fistula in laparoscopic gastrectomy for gastric cancer patients: a propensity score-matched analysis

**DOI:** 10.1007/s00423-022-02692-5

**Published:** 2022-10-01

**Authors:** Kazunori Shibao, Shinsaku Honda, Yasuhiro Adachi, Shiro Kohi, Yuzan Kudou, Nobutaka Matayoshi, Nagahiro Sato, Keiji Hirata

**Affiliations:** Department of Surgery I, School of Medicine, University of Occupational and Environmental Health Japan, 1-1 Iseigaoka, Yahatanishi ward, Kitakyushu, Fukuoka 807-8555 Japan

**Keywords:** Gastric cancer, Energy device, Thermal damage, Pancreatic fistula, Vessel-sealing system

## Abstract

**Background:**

Advanced bipolar devices (ABD; e.g., LigaSure™) have a lower blade temperature than ultrasonically activated devices (USAD; e.g., Harmonic® and Sonicision™) during activation, potentially enabling accurate lymph node dissection with less risk of postoperative pancreatic fistula (POPF) due to pancreatic thermal injury in laparoscopic gastrectomy. Therefore, we compared the efficacy and safety of ABD and USAD in laparoscopic gastrectomy for gastric cancer patients.

**Methods:**

A retrospective cohort study was conducted on patients who underwent laparoscopic distal gastrectomy (LDG) between August 2008 and September 2020. A total of 371 patients were enrolled, and short-term surgical outcomes, including the incidence of ISGPF grades B and C POPF, were compared between ABD and USAD. The risk factors for POPF in LDG were investigated by univariate and multivariate analyses.

**Results:**

A propensity score-matching algorithm was used to select 120 patients for each group. The POPF rate was significantly lower (0.8 vs. 9.2%, *p* < 0.001), the morbidity rate was lower (13.3 vs. 28.3%, *p* < 0.001), the length of postoperative hospitalization was shorter (14 vs. 19 days, *p* < 0.001), and the lymph node retrieval rate was higher (34 vs. 26, *p* < 0.001) with an ABD than with a USAD. There were no mortalities in either group. A multivariate analysis showed that a USAD was the only independent risk factor with a considerably high odds ratio for the occurrence of POPF (USAD/ABD, odds ratio 8.38, *p* = 0.0466).

**Conclusion:**

An ABD may improve the safety of laparoscopic gastrectomy for gastric cancer patients.

## Introduction

According to Globocan, gastric cancer is the fifth-most common malignancy and the third leading cause of cancer-related death worldwide (768,793 deaths, 7.7% of the total) [1]. Despite advances in chemotherapy and radiation therapy in recent years, gastrectomy with regional lymphadenectomy remains the mainstay curative treatment for localized gastric cancer without distant metastasis [2, 3].

However, radical lymphadenectomy recommended in the Japanese guidelines involves the removal of the peripancreatic lymph nodes, which may be accompanied by postoperative pancreatic complications. Although postoperative pancreatic fistula (POPF) is aseptic in the majority of cases, infection of POPF is a significant risk factor for further complications, especially bleeding and mortality [4]. Recently, retrospective and prospective cohort studies have demonstrated that POPF occurs more frequently after laparoscopic gastrectomy (LG) than after open gastrectomy [5–11]. Furthermore, postoperative intra-abdominal infectious complications, such as POPF, prolong the time to the initiation of adjuvant chemotherapy and worsen the long-term outcomes [12, 13]. Therefore, safe and meticulous surgery is essential to prevent POPF for patients undergoing curative gastrectomy.

Advanced bipolar devices (ABD; e.g., LigaSure™) and ultrasonically activated devices (USAD; e.g., Harmonic® and Sonicision™) are the most widely used energy devices in such surgeries [14–18]. Although these instruments have different sealing and cutting mechanisms, they both ensure precise dissection with less bleeding in various surgical procedures. However, ABD and USAD may cause intraoperative collateral heat injury to the pancreas secondary to energized dissection during lymphadenectomy that may result in POPF in LG [6].

We previously reported that the grasping range and repetition of dissection are essential factors that affect the blade temperature of these devices [19]. In particular, USAD with partial tissue bite showed a significantly higher temperature at the blade than ABD. However, our previous report did not include any clinical data. Although many reports have compared the clinical outcomes of conventional surgery using ABD or USAD for various surgical procedures, very few studies for gastrectomy patients have compared the surgical outcomes of these devices [20–22]. Therefore, to compare the efficacy and safety of an ABD with that of a USAD, we retrospectively compared the clinicopathologic characteristics and short-term surgical outcomes, including POPF, of gastric cancer patients undergoing LG. In addition, we performed a risk factor analysis of POPF in LG.

## Patients, materials, and methods

### Study design and inclusion criteria

**Fig. 1:**
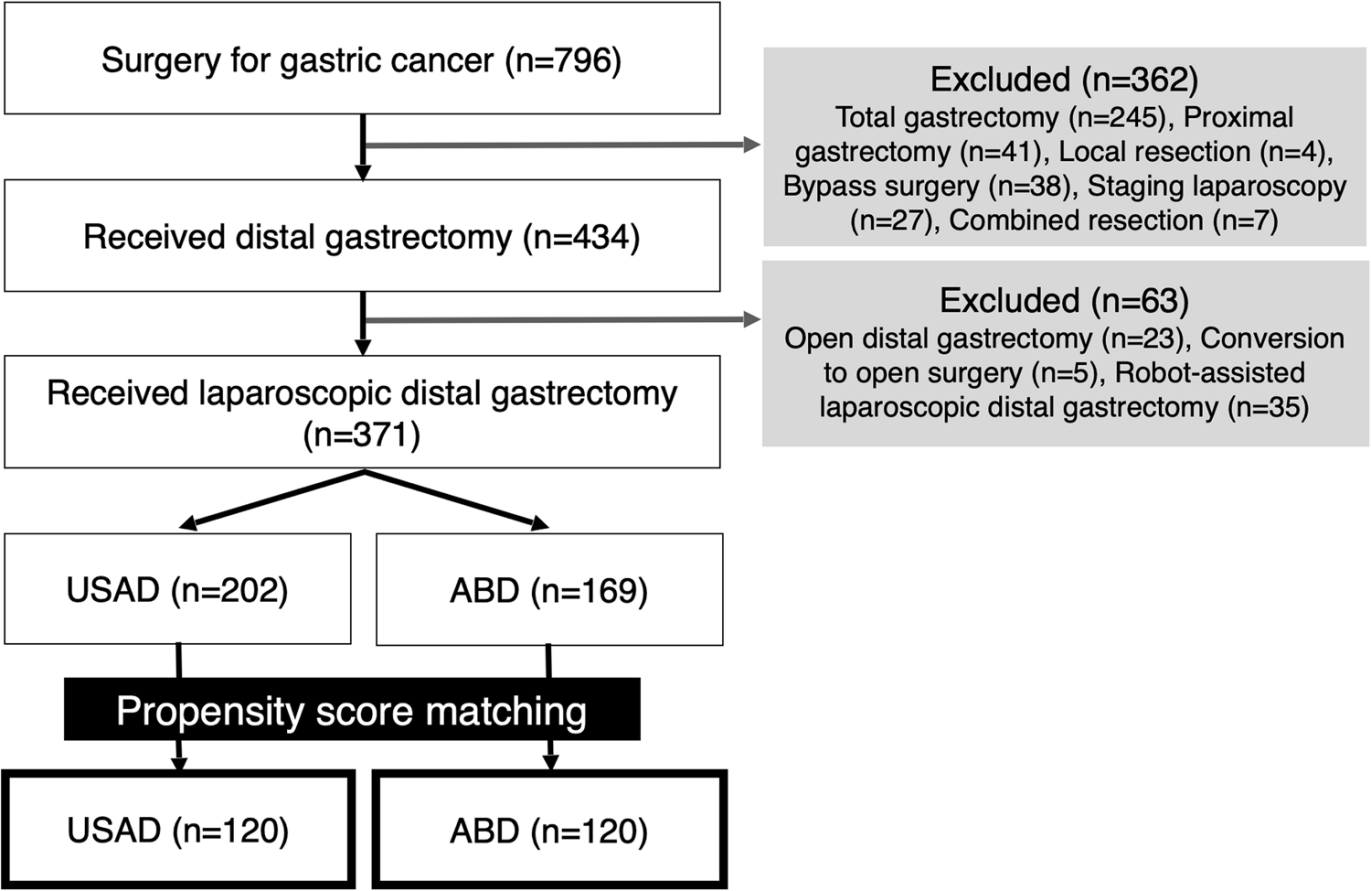
Consort diagram of the participants. Of the 796 consecutive gastric cancer patients, 425 met the exclusion criteria. A PSM algorithm with an optimal caliper was used to select 120 patients in each group

A retrospective cohort study was conducted on patients who underwent laparoscopic distal gastrectomy (LDG) with lymph node dissection to evaluate the short-term surgical outcomes of using an ABD versus a USAD. The inclusion criteria were patients who underwent LDG for gastric cancer at University of Occupational and Environmental Health, Kitakyushu, Japan, between August 2008 and September 2020 (Fig. 1).

The primary endpoint was grades B and C POPF according to the International Study Group for Pancreatic Fistula (ISGPF) classification [23]. The secondary endpoints were postoperative complications, blood loss, operation duration, number of harvested lymph nodes, and length of postoperative hospital stay.

### Data collection

Data on patient characteristics and surgical outcomes were obtained from medical charts. Clinical TNM classification and stage were determined according to the 15th version of the Japanese Classification of Gastric Carcinoma [24]. The extent of lymphadenectomy was classified using the 5th Japanese gastric cancer treatment guidelines [4].

All procedures were performed according to the ethical standards of the respective committees on human experimentation (institutional and national) and complied with the 1964 Declaration of Helsinki and later versions concerning Animal Rights. The Ethics Committee of University of Occupational and Environmental Health, Kitakyushu, Japan, approved this study.

### Surgical procedures

LDG was performed by 10 surgeons, including 3 who were board-certified by the Japanese Society of Endoscopic Surgery. Surgical procedures were standardized for LDG and have been described in detail elsewhere [25–27]. LDG was performed using an ABD (LigaSure™; Medtronic plc, Dublin, Ireland, or Enseal®; Ethicon Endo-Surgery, Cincinnati, OH, USA) or USAD (Harmonic®; Ethicon Endo-Surgery or Sonicision™; Medtronic plc) as the main energy device for dissection, at each surgeon’s preference.

Most of the surgical procedures using ABD were similar to those using USAD, except for the clipless sealing procedure of certain small-caliber vessels. Intracorporal Billroth I reconstruction with functional end-to-end anastomosis or Roux-en-Y reconstruction was performed following distal gastrectomy [28, 29]. A portable drain was routinely inserted at the upper border of the pancreas.

### Definition of POPF

POPF was defined as localized fluid accumulation on computed tomography (CT) and elevated amylase levels in the drain with accompanying abdominal pain and a fever [30]. We considered ISGPF grades B and C POPF to be clinically problematic, requiring some sort of intervention for treatment.

### Statistical analyses

Comparisons were made using the Mann–Whitney *U* test, chi-square test, or Fisher’s exact test, as appropriate. Propensity scores were calculated for all patients based on 11 crucial preoperative clinical variables that might affect the short-term outcome of gastrectomy: Endoscopic Surgical Skill Qualification System for laparoscopic distal gastrectomy (ESSQSG); age; sex; BMI; clinical T, N, and M, tumor size; neoadjuvant chemotherapy; extent of lymphadenectomy; and type of reconstruction [31]. Subsequently, propensity score matching was performed using a 1-to-1 nearest matching algorithm without replacement, with a caliper width equal to 0.2 × the standard deviation of the propensity scores.

Furthermore, the risk factors for POPF were investigated in the propensity score-matched (PSM) cohort by univariate and multivariate analyses. The model included the sex, age, BMI, ESSQSG, cStage, extent of lymphadenectomy, and type of energy device used as possible confounding factors. The multivariate logistic regression models included variables with *P* < 0.01 in univariate analyses, and odds ratios (ORs) with 95% confidence intervals (CIs) were estimated for each variable. The R release 3.6.3 software program was used for all calculations [32]. Significance was set at the *P* < 5% level.

## Results

### Patient selection

A total of 796 consecutive patients underwent surgery for gastric cancer, of whom 425 met the exclusion criteria (Fig. 1). Thus, the remaining consecutive 371 patients were included in the analysis. A PSM algorithm with an optimal caliper was used to select 120 patients in each group.

### Patient characteristics

The background characteristics for all patients and matched patients are shown in Table 1. Before matching, patients in the USAD group had a smaller tumor size (*P* < 0.01), a lower rate of ESSQSG (*P* < 0.01), and a higher rate of Roux-en-Y reconstruction (*P* < 0.01) than the ABD group. After matching, all covariates were well balanced between the ABD and USAD groups.

**Table 1:** Clinicopathology of all patients and matched patients

Patient’s characteristics	All patients		*p* value	Propensity-matched patients		*p* value
	USAD (*n* = 202)	ABD (*n* = 169)		USAD (*n* = 120)	ABD (*n* = 120)	
Sex			0.544			1
Male, *n* (%)	123 (60.9)	109 (64.5)		79 (65.8)	80 (66.7)	
Female, *n* (%)	79 (39.1)	60 (35.5)		41 (34.2)	40 (33.3)	
Age in years			0.299			0.986
Median (range)	70 (33–97)	71 (35–90)		69.3 (33–97)	69.3 (35–88)	
Body mass index in kg/m^2^			0.096			0.808
Median (range)	23 (14.4–35.8)	22 (14.2–45)		22.6 (3.4)	22.4 (4.1)	
Tumor size in mm			< 0.01			0.881
Median (range)	30 (10–110)	35 (10–150)		36.9 (10–110)	36.5 (10–120)	
Tumor pathology			0.172			0.897
Differentiated, *n* (%)	104 (51.5)	90 (53.3)		67 (55.8)	65 (54.2)	
Undifferentiated, *n* (%)	98 (48.5)	79 (46.7)		53 (44.2)	55 (45.8)	
cT category			0.814			0.909
1/2/3/4, *n*	101/41/38/23	78/36/15/40		60/29/13/18	59/26/13/22	
cN category			0.072			1
0/ + , *n*	131/71	92/77		72/48	73/47	
cM category			0.253			0.37
0/1, *n*	200/2	164/5		119/1	116/4	
cStage			0.053			0.534
I/IIA/IIB/III/IV, *n*	119/17/12/52/2	85/28/5/47/4		70/16/2/31/1	69/15/5/27/4	
Neoadjuvant chemotherapy			1			1
Yes, *n* (%)	0 (0)	7 (4.1)		0 (0)	4 (3.3)	
No, *n* (%)	202 (100)	162 (95.9)		120 (100)	116 (96.7)	
ESSQSG-certificated surgeon			< 0.01			1
Yes, *n* (%)	62 (30.7)	101 (59.8)		54 (45)	54 (45)	
No, *n* (%)	140 (69.3)	68 (40.2)		66 (55)	66 (55)	
Reconstruction			< 0.01			1
Birroth I, *n* (%)	143(70.8)	88 (52.1)		78 (65)	80 (66.7)	
Roux-en-Y, *n* (%)	59 (29.2)	81 (47.9)		42 (35)	40 (33.3)	
Extent of dissection			1			0.691
< D2, *n* (%)	120 (59.4)	100 (59.2)		72 (60)	76 (63.3)	
D2, *n* (%)	82 (40.6)	69 (40.8)		48 (40)	44 (36.7)	

*ESSQSG* Endoscopic Surgical Skill Qualification System for laparoscopic distal gastrectomy

### Short-term outcomes and postoperative complications

Energy devices and surgical outcomes, including details concerning postoperative complications before and after matching, are shown in Table 2. Before matching, POPF was more frequent in the USAD group than in the ABD group (6.4 vs. 0.6%, *P* <0.001). The overall postoperative complication rate for ISGPF grade B was not significantly different between the USAD and ABD groups (18.3 vs. 12%, *P*=0.183), but the USAD group had more blood loss (50 vs. 30 g, *P* < 0.001) and fewer harvested lymph nodes (24 vs. 32, *P* < 0.001) thanthe ABD group. In the matched analysis, POPF was significantly more frequent in the USAD group than in the ABD group (9.2 vs. 0.8%, *P* < 0.001). The USAD group had fewer harvested lymph nodes (26 vs. 34, *P* < 0.001) than the ABD group, as well as longer postoperative hospitalization (19 vs. 14 days, *P* < 0.001) and higher overall postoperative morbidity rates (28.3 vs. 13.3%, *P* <0.001). There was no marked difference in the types of postoperative complications between the USAD and ABD groups, except for POPF. No 90-day mortality was observed in either group.

**Table 2:** Energy devices and surgical outcomes before and after matching

Surgical outcomes	All patients		*p* value	Propensity-matched patients		*p* value
	USAD (*n* = 202)	ABD (*n* = 169)		USAD (*n* = 120)	ABD (*n* = 120)	
Blood loss in g, median (range)	50 (0–1100)	30 (0–840)	< 0.01	98 (0–1100)	68 (0–840)	0.063
Operation duration in min, median (range)	280 (152–1350)	292 (156–1229)	0.335	309 (168–1350)	300 (156–1229)	0.571
Number of dissected lymph nodes, median (range)	24 (2–56)	32 (2–75)	< 0.01	26 (2–56)	34 (2–75)	< 0.01
Postoperative hospitalization, median (range)	13 (8–85)	12 (7–259)	0.126	19 (8–85)	14 (7–49)	0.003
90-day mortality	2 (1)	0	1	0	0	1
Overall postoperative morbidity, *n* (%)	37 (18.3)	21 (12)	0.183	34 (28.3)	16 (13.3)	0.007
POPF, ISGPF grades B and C, *n* (%)	13 (6.4)	1 (0.6)	< 0.01	11 (9.2)	1 (0.8)	0.005
Intraabdominal abscess, *n* (%)	0	1 (0.6)	0.456	0	0	1
Anastomotic leakage, *n* (%)	7 (3.47)	2 (1.2)	0.19	7 (5.8)	1 (0.8)	0.066
Anastomotic stricture, *n* (%)	6 (3)	4 (2.4)	0.76	6 (5)	3 (2.5)	0.5
Lympholea, *n* (%)	1 (0.5)	1 (0.6)	1	1 (0.8)	1 (0.8)	1
Small bowel obstruction, *n* (%)	1 (0.5)	0	1	0	0	1
Ileus, *n* (%)	1 (0.5)	5 (3)	0.097	1 (0.8)	4 (3.3)	0.37
Pneumonia, *n* (%)	4 (2)	4 (2.4)	1	4 (3.3)	4 (3.3)	1
Others	9 (4.5)	3 (1.8)	0.247	4 (3.3)	2 (1.7)	1

### Risk factors for POPF

To identify risk factors for POPF, a risk factor analysis was performed. The results of univariate and multivariate analyses of clinical factors are summarized in Table 3. In univariate analyses, POPF was significantly associated with an older age (OR=0.69, *P* <0.001), higher BMI (OR=4.6, *P* <0.001), higher cStage (OR=0.716, *P* <0.001), greater extent of lymph node dissection (OR=1.64, *P* <0.001), and the type of energy device used for dissection (USAD/ABD, OR=9.65, *P*<0.001) but not with the sex (*P* = 0.358) or surgeon qualification (*P* = 0.338). A multivariate analysis indicated that the type of energy device was the only independent risk factor with a considerably high OR for the occurrence of grades B and C POPF (USAD/ABD, OR 8.38, *P* = 0.047).

**Table 3:** Risk factor analysis for ISGPF grades B and C postoperative pancreatic fistula according to ISGPF classification in the matched cohort (*n* = 240)

Variables	Univariate analysis		Multivariate analysis	
	Odds ratio (95% CI)	*p* value	Odds ratio (95% CI)	*p* value
Sex (M/F)	2.092 (0.434–109.89)	0.358		
Age in years (> 71/ < 71)	0.69 (0.19–2.51)	< 0.01	0.763 (0.195–2.99)	0.697
Body mass index in kg/m^2^ (> 22.5/ < 22.5)	4.6 (0.956–22.1)	< 0.01	3.95 (0.805–19.4)	0.09
ESSQSG-certified surgeon (yes/no)	0.51 (0.129–2.02)	0.338		
cStage (I/ > II)	0.716 (0.202–2.54)	< 0.01	0.814 (0.186–3.79)	0.821
Lymph node dissection (D2/ < D2)	1.64 (0.463–5.84)	< 0.01	1.44 (0.332–6.26)	0.625
Energy device (USAD/ABD)	9.65 (1.2–77.4)	< 0.01	8.38 (1.03–68)	0.047

Odds ratio was calculated using the latter variable as a reference, e.g., male to female

*ISGPF* International Study Group for Pancreatic Fistula, *OR* odds ratio, *CI* confidence interval, *USAD* ultrasonically activated device, *ABD* advanced bipolar device, *ESSQSG* Endoscopic Surgical Skill Qualification System for laparoscopic distal gastrectomy

## Discussion

When considering the safety and feasibility of LG, it is vital to assess the risks of POPF formation. PF after gastrectomy is thought to be caused by intraoperative injury to the pancreas. Possible causes of pancreatic injury during LG include thermal injury from energy devices, compression from forceps, accidental injury from anatomical variations in the shape and location of the pancreas, and pancreatic steatosis [6, 7, 19, 33–38]. Among them, heat injury caused by an ultrasonic device is a significant cause of pancreatic damage during LG [6, 33, 39]. In this retrospective study, we demonstrated that LDG with peripancreatic lymphadenectomy using an ABD was associated with a lower rate of POPF and morbidity than that using a USAD for gastric cancer surgery. The incidence of clinically relevant POPF (ISGPF grades B and C) of 0.8% in the ABD group was significantly lower than that of 9.2% in the USAD group (Table 2). These results are comparable to those reported in a recent study of laparoscopic surgery performed at a high-volume center in Japan, which found a 1.0–8.9% incidence of pancreatic fistula [5–8, 10, 11]. Other advantages of an ABD included a lower overall morbidity rate, more lymph nodes retrieved, and a shorter hospital stay. We also revealed in a multivariate analysis of a PSM cohort that only the usage of a USAD was a significant independent risk factor for POPF in LDG.

ABD create a vessel seal by applying bipolar electrosurgical radiofrequency energy to vessels interposed between the jaws of the device [15]. In contrast, USAD use high-frequency mechanical energy to disrupt hydrogen bonds in tissues and denature proteins [14]. Each device has its own advantages and disadvantages due to differing thermal profiles and sealing and cutting mechanisms. We previously reported that repeated dissection of energy devices with minimal cooling time results in high blade and jaw temperatures proportional to the incision distance. In particular, the USAD with partial tissue bite showed a significantly higher temperature at the blade (341 ± 28.3 °C) than that with the ABD (95.6 ± 5.5 °C) [19]. Due to the relatively low temperature of the activated blade and limited lateral thermal spread (0.9 mm beyond the tissue within the jaws), surgeons can use an ABD to dissect lymph nodes close to the pancreas and blood vessels, thereby minimizing the risk of damaging delicate adjacent structures while ensuring that the target lymph node can be completely removed [15, 19]. In addition, compared to a USAD, an ABD is more effective at achieving hemostasis and produces less surgical smoke and mist, as shown by the present results and previous reports, thereby improving the surgical visibility and lymph node clearance [20, 21]. However, another author believed that an ABD was not suitable for the precise surgical maneuvers required for operations such as gastrectomy with lymph node dissection for gastric cancer [22]. As reported earlier, an ABD requires some technical proficiency but can be used to perform definitive lymph node dissection more safely than a USAD [20]. Regardless of which energy device is used, surgeons should understand the thermal profile of the device and avoid inappropriate activation during suprapancreatic lymph node dissection in order to prevent thermal injury to the pancreas in LDG. The Society of American Gastrointestinal and Endoscopic Surgeons (SAGES) developed the Fundamental Use of Surgical Energy™ (FUSE) program to meet the need for increased education and training in the principles and properties of operating electrosurgical instruments safely [16]. The FUSE program facilitate the understanding and application of these fundamental skills concerning energy devices and thereby promote the safe care of all patients undergoing surgery.

There have been many reports comparing the outcomes of procedures using ABD with those of conventional surgery for various surgical procedures [21, 22, 40, 41]. However, only a limited number of studies have compared the surgical outcomes of ABD and USAD. No significant difference in short-term surgical results between ABD and USAD was reported in laparoscopic colorectal resection, laparoscopic sleeve gastrectomy, or Roux-en-Y gastric bypass [42–44]. In thyroidectomy, a previous study found no marked difference between the ABD and USAD groups in the efficacy or surgical outcomes of total thyroidectomy [18]. Another group reported that an ABD was inferior to a USAD in terms of the operative time [45]. Notably, Kim et al. reported that in LDG with extended lymph node resection among 186 patients with gastric cancer, the ABD had advantages over the USAD with regard to operative time, degree of postoperative pain, time for drain removal, and length of hospital stay [46]. However, their study did not show any advantage of an ABD over a USAD in terms of safety. In the present study, the ABD group exhibited a lower rate of POPF and mortality, more dissected lymph nodes, and a shorter hospital stay than the USAD group in LDG with lymphadenectomy for 240 gastric cancer patients. Thus, the present report is the largest series comparing the short-term surgical results of an ABD and a USAD in LDG and the first to demonstrate the safety of an ABD over a USAD in reducing POPF and mortality in LDG.

Several limitations associated with the present study warrant mention. First, this study employed a retrospective, single-center, non-randomized design. We compared the short-term outcomes of ABD and USAD after matching two groups using propensity scores to reduce any bias in the comparison. However, there may have been unknown confounders, including the potential for selection biases, so the overall results should be interpreted cautiously. Second, the superiority of an ABD with regard to oncological outcomes is not yet conclusive, as long-term surveillance has not been conducted. Third, other factors, such as advances in individual surgical techniques, might have influenced the improvement in surgical outcomes. Because LDG with the ABD was a new procedure for some surgeons at the start of the study, the ABD group included cases in which the surgeon was using the device for the first time. Thus, even in single-center trials, it can be very difficult to ensure that all doctors have the same level of experience and proficiency. However, this study is the largest series comparing the short-term surgical results of an ABD and a USAD for LDG and is the first to demonstrate the safety of an ABD over a USAD in reducing POPF and mortality following LDG.

## Conclusion

In conclusion, the present study suggested that the ABD may be a safe and beneficial energy device for LDG, as the ABD was significantly more advantageous than the USAD in terms of the POPF and morbidity rates, number of lymph nodes retrieved, and postoperative hospital stay. To safely perform LG with lymph node dissection in patients with gastric cancer, the mechanism underlying the heat conductance of energy devices needs to be better considered, which may reduce the incidence of POPF. However, no matter what kind of energy device is used, surgeons need to be careful to avoid damaging normal tissues during surgery and ensure patient safety. Further studies involving multiple centers, a larger population, and a randomized controlled trial with long-term surveillance are needed to establish solid evidence concerning the safety of ABD.

## Abbreviations

ABD: Advanced bipolar device
USAD: Ultrasonically activated devices
POPF: Postoperative pancreatic fistula
LG: Laparoscopic gastrectomy
LDG: Laparoscopic distal gastrectomy
CT: Computed tomography
SNC: Sonicision™ Shears
LSM: LigaSure™ Maryland jaw
PSM: Propensity score-matched
OR: Odds ratio
CI: Confidence interval
ISGPF: International Study Group for Pancreatic Fistula
ESSQSG: Endoscopic Surgical Skill Qualification System for laparoscopic distal gastrectomy
